# Face processing in animal models: implications for autism spectrum disorder

**DOI:** 10.3389/fnins.2024.1462272

**Published:** 2024-08-09

**Authors:** Paola Sgadò, Alessandra Pross, Jacopo Lamanna, Alice Adiletta

**Affiliations:** ^1^Center for Mind/Brain Sciences, University of Trento, Rovereto, Italy; ^2^Center for Behavioral Neuroscience and Communication (BNC), Vita-Salute San Raffaele University, Milan, Italy; ^3^Faculty of Psychology, Vita-Salute San Raffaele University, Milan, Italy

**Keywords:** face detection, social orienting, domestic chick, neurodevelopmental disorders, valproic acid

## Abstract

Processing facial features is crucial to identify social partners (prey, predators, or conspecifics) and recognize and accurately interpret emotional expressions. Numerous studies in both human and non-human primates provided evidence promoting the notion of inherent mechanisms for detecting facial features. These mechanisms support a representation of faces independent of prior experiences and are vital for subsequent development in social and language domains. Moreover, deficits in processing faces are a reliable biomarker of autism spectrum disorder, appearing early and correlating with symptom severity. Face processing, however, is not only a prerogative of humans: other species also show remarkable face detection abilities. In this review, we present an overview of the current literature on face detection in vertebrate models that could be relevant to the study of autism.

## Introduction

Faces convey a great amount of socially relevant information related to emotional and mental states, identity, and intention. Processing facial information is crucial for social and cognitive development (Wagner et al., 2013), so much so that newborns within an hour of birth are already biased to orient to faces and prefer them over any other stimulus (Johnson et al., 1991; Valenza et al., 1996). This early preference is even observed towards schematic geometric (face-like) patterns resembling a face (three black squares organized as an upside-down triangle; Goren et al., 1975; Johnson et al., 1991) and might already be present during the last gestational trimester (Reid et al., 2017). Although still a matter of debate, evidence suggests the presence of a specialized cognitive system present since the first moments of life. Some authors have proposed a two-process model supporting face processing to explain newborns’ preference for real and schematic faces (Johnson et al., 1991; Morton and Johnson, 1991). According to this model, the inherent predisposition of newborns to visually attend to faces and schematic face-like stimuli is guided by a rudimental representation (CONSPEC; see Morton and Johnson, 1991), which can drive their preference for simple, geometrical characteristics of these stimuli (oval bounded area, top-heavy featural pattern, and positive contrast; see Johnson, 2005). Neuropsychological studies indicating residual face processing abilities in patients with visual cortical impairments and electrophysiological studies suggest the involvement of a visual system supporting fast and coarse (based on low spatial frequencies) facial detection localized in subcortical areas such as the superior colliculus, the pulvinar, and the amygdala (Johnson, 2005). As development proceeds, the predisposition is complemented by a second adaptive mechanism (termed CONLEARN; see Morton and Johnson, 1991) relying on preferential exposure to faces to refine and adapt the cortical representations of faces and to ascribe identity. Other authors have proposed alternative hypotheses to explain the early preference responses to face and face-like stimuli based on intrinsic visual properties of the stimuli, such as the stimulus energy (Kleiner, 1987) or the structural properties of the stimuli (for example the presence of a “top-heavy” configuration, Turati et al., 2002; Simion and Di Giorgio, 2015) that may drive the preference for faces in human newborns without being related to *“facedness.”* Moreover, a recent study investigating face processing in newborns challenges the hypothesis that the preference for face-like stimuli is mainly mediated by subcortical structures, demonstrating a clear activation of cortical structures upon exposure to upright face-like compared to inverted or scrambled stimuli (Buiatti et al., 2019).

Several studies indicate that the visual processing of facial information is significantly compromised in neurodevelopmental disorders (Collin et al., 2013). Schizophrenia patients show deficits in face detection (Chen et al., 2008; Romagnano et al., 2022), face identity (Russell et al., 2024), and facial emotion recognition (see for a review Bortolon et al., 2015). Moreover, disruptions in face processing are one of the earliest indicators of social deficits in autism spectrum disorders (ASD) and play a key role in the pathophysiology of these disorders (Johnson, 2014; Johnson et al., 2015; Pavlova et al., 2017). Infants at risk of ASD often exhibit impairments in processing face and eye-gaze direction, further emphasizing the significance of these social orienting mechanisms as early biomarkers for ASD.

However, processing facial information is not just a prerogative of humans; several other animal species possess remarkable face recognition abilities.

Like humans, infant non-human primates prefer faces (Pascalis and Kelly, 2009; Pascalis et al., 2021). Among mammals, sheep and other ungulate species show sophisticated face recognition abilities (Proops and McComb, 2012; Towler et al., 2019); dogs have been shown to respond specifically to their owners’ faces and recognize their facial emotional expressions (Barber et al., 2016; Cuaya et al., 2016). Several avian species possess remarkable face processing abilities (Brecht et al., 2017; Suwandschieff et al., 2023), including pigeons and domestic chicks, where neural correlates of face discrimination have been investigated (Clark et al., 2022; Kobylkov et al., 2024). Fish also rely on visual information in the head and face regions to drive their social affiliative responses (Karplus and Algom, 1981; Wang and Takeuchi, 2017; Nunes et al., 2020). Finally, individual recognition based on facial features has been described also in paper wasps and honeybees (Avarguès-Weber et al., 2018; Tibbetts et al., 2021).

Face processing comprises multiple functional components, including visual processing, facial identity analysis, and facial emotion expression recognition. We focus here on face detection, defined as the ability to detect the presence of faces in the visual scene based on first-order information (i.e., basic spatial properties of facial features). This form of visual discrimination is the simplest and earliest form of face processing, and it is more likely to be ubiquitous across species.

Moreover, the similarities in face processing observed in different species suggest a common origin that could be exploited to investigate the underlying neurobiological mechanisms and to elucidate the earliest expression of social deficits in neurodevelopmental disorders such as ASD. This review presents an overview of the current literature on face detection in vertebrate models potentially relevant to the study of ASD.

## Evidence of face processing in newborn vertebrates

A significant contribution to the knowledge about face detection comes from the extensive literature on face processing development in non-human primates (see Pascalis et al., 2021 for a review). Infant primates, including humans, exhibit a strong preference for faces. Cross-fostering and restricted social experience experiments in rhesus (*Macaca mulatta*) and Japanese monkeys (*Macaca fuscata*) indicate differences in the ability of these two species to respond to the faces of other species. Japanese macaques raised without face experience for several months maintained their preferences for both monkey and human faces, showing an innate response to faces independent of the species (Sugita, 2008). After limiting their visual experience to human or monkey faces, their preference was tuned toward the predominant faces they were exposed to, demonstrating a strong influence of experience on the innate representation of faces (Sugita, 2008, 2009). Rhesus macaques, instead, express a strong preference for faces of their own species already at 3 months of age (Fujita, 1987, 1993). Subsequent studies on young infants (3 weeks old) rhesus macaques raised without face experience also supported the idea of an early coarse representation of faces that is quickly refined to the own species due to exposure (Simpson et al., 2017). This data supports the existence, also in monkeys, of hard-wired mechanisms providing an early representation of faces independent of experience. These mechanisms are then fine-tuned by experience according to the predominant face stimuli found in the environment.

In addition to Japanese macaque monkeys, domestic chicks (Rosa-Salva et al., 2010), and land tortoises (Versace et al., 2020) show innate face preference responses similar to those observed in human newborns.

Research involving domestic chicks (*Gallus gallus domesticus*) offers a convenient method for regulating the animals’ visual exposure prior to hatching by maintaining the eggs and the hatchlings in complete darkness. Unlike many mammalian species, dark rearing does not compromise the chicks’ visual system’s development. Moreover, domestic chicks are a precocial species: the hatchlings already possess a mature visual system, can immediately and efficiently explore the environment, and are strongly driven to social interaction. Thanks to these advantages, studies on domestic chicks have demonstrated the presence of innate representations of visual stimuli important for the animals’ survival, including faces and face-like schematic configurations (Rosa-Salva et al., 2010; Rosa Salva et al., 2012), biological motion (Vallortigara et al., 2005; Zanon et al., 2024), and animacy (Rosa Salva et al., 2015). As for face detection, a series of experiments showed that visually inexperienced newborn chicks are spontaneously driven to approach a schematic stimulus representing a face compared to several other stimuli sharing the same top-heavy configuration but lacking the “*facedness”* property (Rosa-Salva et al., 2010). Subsequent studies demonstrated the sensitivity of the chicks to the reversal of contrast polarity and a right hemisphere advantage for the detection of faces (Rosa Salva et al., 2012) similar to what was shown in human neonates (Farroni et al., 2005; Buiatti et al., 2019). Overall, domestic chicks seem to express orienting responses to visual stimuli equivalent to those observed in developmental human studies, advocating this flexible animal model to investigate the neurobehavioral bases of face detection.

## Neural correlates of face detection

Visual object recognition in mammals is mediated by a series of recurrent, hierarchically, and topographically organized cortical circuits, often referred to as the ventral visual processing stream. Visual information is sent from the primary visual cortex (V1) to the extra-striate occipital visual areas and converges in the ventral part of the temporal cortex in high-level visual areas. The simplest aspect of face processing, which involves detecting the presence of faces in the visual scene, requires extracting basic features of the stimulus independent of contexts and viewpoints. Face detection has been hypothesized to involve a different hierarchical visual pathway than the recognition of other objects and to be supported by cortical face-selective areas (Kanwisher et al., 1997; McCarthy et al., 1997). Face-selective regions in the brain have been described in the ventrolateral aspects of the occipital and temporal cortex (see for review Duchaine and Yovel, 2015). These areas respond preferentially to faces, being active significantly more in response to faces than other non-face visual stimuli (objects, places, body parts, and letters). The occipital face area (OFA; Gauthier et al., 2000), an early visual area in the inferior occipital gyrus, and the face-selective region in the superior temporal sulcus (fSTS; Hoffman and Haxby, 2000) produce an initial representation of faces based on first-order elements, i.e., eyes, nose and mouth, but lack sensitivity to their spatial configuration (Pitcher et al., 2007; Liu et al., 2010). The most robust face-selective activation is observed in the fusiform gyrus of the temporal lobe, named the fusiform face area (FFA; Kanwisher et al., 1997; McCarthy et al., 1997). Numerous studies have indicated that FFA is activated in response to face stimuli rather than their visual features (Liu et al., 2010) and that it exhibits selective activation in response to different types of facial stimuli, including photographs, drawings, and depictions of animal faces (Tong et al., 2000). Moreover, the neural representation within the FFA is sensitive to the “inversion effect” (originally unveiled through the “Thatcher illusion” by Thompson, 1980), which causes a disproportionate drop in recognition of upside-down (inverted) faces compared to upright faces not observed for inverted objects (Yovel and Kanwisher, 2005).

A recent report found evidence of cortical involvement in face processing at birth, similar to the face-selective areas observed in the adult brain (Buiatti et al., 2019). Using high-density EEG and measuring frequency-tagged signals in newborns exposed to upright face-like, inverted, and scrambled face stimuli, the study found a stronger response to face-like stimuli in cortical structures along the occipitotemporal pathway, similar to those observed in adults (Buiatti et al., 2019).

In addition to the face-selective areas in the visual cortex, an extended network of additional face processing areas has been described as involved in identity recognition, facial expression and emotional processing (Haxby et al., 2000; Calder and Young, 2005). These include lateral prefrontal cortex regions that are involved in both featural and configural processing of faces and provide a top-down control to the temporal cortex (Heekeren et al., 2004; Renzi et al., 2013).

Non-human primate research has also been fundamental in investigating the neural bases of face perception (Tsao and Livingstone, 2008; Rossion and Taubert, 2019). Face-selective areas, called face patches, have been described in macaque monkeys (Tsao et al., 2008), vervet monkeys (Zangenehpour and Chaudhuri, 2005), marmosets (Hung et al., 2015), and chimpanzees’ (Parr et al., 2009) inferotemporal (IT) cortex.

In parallel to discovering face-selective areas in the temporal cortex of humans and non-human primates, several studies demonstrated that a subcortical visual pathway largely mediates face perception (Johnson et al., 2015; Almasi and Behrmann, 2021). It is widely recognized that newborns have the ability to orient themselves toward faces and stimuli that resemble faces. Considering the poor maturation of the cerebral cortex in the first months after birth, many authors have suggested that subcortical visual pathways may support the immature cortical structures during this time (Johnson, 2005). Evidence confirms this hypothesis, including recent studies showing a monocular advantage in infants’ and adults’ face processing (Almasi and Behrmann, 2021; Dalrymple et al., 2021) and suggesting the engagement of subcortical visual pathways. This evolutionarily conserved visual system includes part of the retinocollicular system, the amygdala, the lateral geniculate nucleus, the pulvinar, and the superior colliculus. In addition to studies in infants, face-selective activation has also been demonstrated in subcortical regions of the adult brain, independent of emotional expression, for example, in the amygdala and the superior colliculus (Mende-Siedlecki et al., 2013). Recent studies in monkeys have demonstrated that a specific population of neurons in the superior colliculus responded stronger and faster to the face-like than non-face patterns (Le et al., 2020), elucidating some of the subcortical neural correlates involved in face detection. To reconcile with recent reports of cortical areas in the occipitotemporal cortex participating in newborns’ face detection (Buiatti et al., 2019), studies have suggested a substantial link between the subcortical and the cortical circuits to support face processing in early development (Pessoa and Adolphs, 2010).

A recent report investigated face-selective neurons also in domestic chicks (Kobylkov et al., 2024). The authors examined the response of a group of neurons in the nidopallium, a region of the chick brain believed to be homologous to the human prefrontal cortex, of face-naïve young domestic chicks exposed to schematic face-like stimuli. Using single-cell recordings, Kobylkov et al. (2024) demonstrated the presence of face-selective neurons in the caudolateral nidopallium of domestic chicks that respond significantly stronger to upright face-like stimuli compared to other configurations (inverted, asymmetric, or frequency-filtered facial stimuli) or to face parts (Kobylkov et al., 2024). Moreover, the authors demonstrated that face-selectivity in this neuronal population emerges independently of previous visual experience, supporting the hypothesis of an innate system for face detection in domestic chicks (see for a review Kobylkov and Vallortigara, 2024). Interestingly, face selectivity has been described in the prefrontal cortex of both humans (Heekeren et al., 2004; Renzi et al., 2013) and non-human primates (Chan et al., 2016; Schaeffer et al., 2020) demonstrating, once more, the strong translational value of this animal model.

## Face detection in neurodevelopmental disorders: an animal model perspective

Face processing deficit is the most important early impairment in ASD and one of the most reliable findings in the literature. Several studies have examined face processing abilities in ASD (see for a review Campatelli et al., 2013; Bi and Fang, 2017), documenting behavioral differences at different levels: facial expression recognition (Uljarevic and Hamilton, 2013; Lozier et al., 2014; Loth et al., 2018), individual identity recognition (Weigelt et al., 2012; Minio-Paluello et al., 2020), visual attention to faces (Klin et al., 2002; Pelphrey et al., 2002; Riby and Hancock, 2009; Chita-Tegmark, 2016; Reisinger et al., 2020), and face perception (Carver and Dawson, 2002; Klin et al., 2002). Deficits in visual attention and face detection seem to characterize ASD from very early in life: while face detection deficits in ASD have been reported more consistently in older children and adults (Webb et al., 2017; Bathelt et al., 2022), newborns with increased ASD risk also show reduced responses to face-like stimuli already at birth (Di Giorgio et al., 2016, 2021; Bradshaw et al., 2020). In addition, neurophysiological and neuroimaging studies have shown altered activation patterns in cortical brain areas associated with face detection (Shephard et al., 2020; Tye et al., 2022). Hypoactivation of FFA during tasks involving face perception appears to be the most consistently observed functional abnormality in ASD (Schultz et al., 2000, 2003; Schultz and Klin, 2002; Nickl-Jockschat et al., 2015; Wang et al., 2024). Other subcortical face processing networks exhibit functional alterations and modified connectivity in individuals with ASD (Kleinhans et al., 2011). For example, the amygdala displays atypical activation in ASD (Kliemann et al., 2012; Philip et al., 2012; Rutishauser et al., 2013; Wang and Li, 2023); aberrant activation in ASD was also described in the pulvinar and the superior colliculus (Kleinhans et al., 2011; Huang et al., 2022).

Despite the advancement in the understanding of brain connectivity and functional changes related to face processing abnormalities in ASD, the neurobiological bases of these dysfunctions are still not well understood. Given the high heterogeneity of the symptoms and neurobiological alterations associated with ASD (Lamanna and Meldolesi, 2024), as well as the adaptative changes occurring in the developing brain to compensate for the deficits, understanding the mechanisms behind face processing abilities in ASD requires developmental studies. So far, very few studies have addressed the developmental aspect of these deficits, i.e., what are the mechanisms behind the lack of preferential attention to faces in individuals with ASD from early developmental stages? Some authors (Dawson et al., 2005) have proposed that face processing deficits are mediated by underlying impairment in social motivation, resulting in the failure to attend to socially relevant stimuli and, therefore, limiting social development. Others (Johnson, 2005) have suggested that in individuals with ASD, the disruption of the subcortical face processing pathway is a primary cause of impaired social orienting mechanisms, which in turn compromises exposure to faces and, therefore, the development of typical social abilities.

Given human neonatal studies’ limits and ethical constraints, together with the pervasive nature of face processing in vertebrates, adopting a developmental approach to studying face detection mechanisms with a comparative perspective in animal models of ASD may represent a valuable strategy to investigate these deficits.

The development of highly efficient and precise genetic tools has significantly spurred research in modeling human disorders in non-human primates, including in the context of autism (Zhao et al., 2018). A recent study by Zhou et al. (2019) analyzed the effect of a mutation in the SH3 and ankyrin repeat domains 3 (*SHANK3*) gene, a highly penetrant, monogenic risk factor for ASD, on Cynomolgus macaque monkeys (*Macaca fascicularis*) visual attention to social and nonsocial stimuli. Using images of faces, objects, and faces with threatening or neutral expressions, the authors found a reduced fixation time and increased aversion to the images in the mutant monkey compared to the controls despite the low number of mutant monkeys analyzed (5 mutants, 1 female). Another study was recently conducted (Zhao et al., 2019) investigating attentive behavior towards faces in juvenile macaques (*Macaca fascicularis*) exposed to valproic acid (VPA), an anticonvulsant known to interfere with the development of the social brain and increase the risk of developing ASD in humans (Christensen et al., 2013). VPA’s mechanism of action involves its direct inhibition of histone deacetylases (HDACs), interfering with normal chromatin deacetylation and disrupting the transcription of multiple ASD-associated genes (Meng et al., 2022; Guerra et al., 2023; Krueger et al., 2024; Zarate-Lopez et al., 2024). Using eye-tracking analysis to measure the animals’ attention to faces or scenes containing conspecifics, the authors found that juvenile monkeys exposed to VPA attended to non-social stimuli significantly more than their control siblings (Zhao et al., 2019). Studies have also been carried out on models of ASD in marmoset monkeys (*Callithrix jacchus*) exposed to VPA. Nakagami et al. (2022) examined the visual attention of juvenile and adult marmosets exposed to VPA and found a reduction in the time spent gazing at other conspecifics already at 15–19 weeks.

Despite the limited reports of direct face detection testing in monkeys, the evidence accumulated so far is promising, and the potential for investigating the neural correlates and neurobiological mechanisms underlying the reported deficits in social preference is encouraging (Watson and Platt, 2012; Bauman and Schumann, 2018; Katsnelson, 2018; Zhao et al., 2018).

In addition to human newborns and monkeys, several other vertebrate species possess remarkable face detection abilities. In domestic chicks, younglings express striking orienting responses to a broad spectrum of visual representations of the typical appearance and features associated with living beings. Several studies have investigated the behavioral and neurobiological bases of these abilities in domestic chicks, including extensive investigation of their innate preferences for human faces and face-like stimuli (see for a review Di Giorgio et al., 2017). Thanks to their precocious and strong social response to visual cues, domestic chicks have also attracted attention as model organisms to investigate other behavioral and neurobiological mechanisms relevant to autism (Csillag et al., 2022; Matsushima et al., 2024). Exposure to the anticonvulsant VPA impairs the chicks’ ability to orient to the appearance of a stuffed hen (Sgadò et al., 2018) to motion cues typical of animate agents (Lorenzi et al., 2019) and, most interestingly, to face-like configurations similar to those that bias the attention of human neonates (Adiletta et al., 2021). Interestingly, neurobiological mechanisms potentially relevant for impairments in face processing are starting to be investigated (Adiletta et al., 2022).

## Discussion

Despite a consensus on the significance of face processing in social development and ASD, the nature and origin of the face processing deficits and the underlying neurobiological mechanisms are still unclear. Thanks to the pervasive nature of face processing in vertebrates, employing a comparative approach to study the development of face detection mechanisms in animal models of ASD may shed light on these mechanisms.

Many studies investigating the neurobiological bases of ASD in animal models are focused on rodents, especially mice, thanks to their genetic and neuroanatomical homology to humans. However, given the prolonged postnatal development of the visual system and the difficulty in analyzing visually mediated early behavioral responses, research into face processing in rodents has been limited (Schnell et al., 2019; Watanabe et al., 2022). However, both rats and mice have remarkable visual perception abilities (Djurdjevic et al., 2018) that could be exploited to further investigate face processing deficits in rodent ASD models.

Prioritizing species with early visual perceptual skills that replicate many of the features of face processing in humans, readily testable at postnatal developmental stages, may be instrumental in providing the key to clarifying the nature and origin of these mechanisms and their role in atypical social development. Very few studies have explored this approach.

The recent development of highly efficient and precise genetic tools, such as CRISPR/Cas9 and TALEN (transcription activator-like (TAL) effector nucleases), makes exploring transgenic strategies in several species possible, including the domestic fowl. Since the first transgenic chick was generated (Salter et al., 1987), much effort has been devoted to developing strategies to induce genetic modifications and improve germline transmission in domestic chicks (see for a review Sid and Schusser, 2018). CRISPR/Cas9-mediated genome editing has been previously employed in domestic chicks to generate somatic mutations in early embryos used for developmental studies (Gandhi et al., 2017), but only recently have stable Cas9-expressing chicken lines been generated (Rieblinger et al., 2021), opening new perspectives to yield loss-of-function mutations in this species, including those to model ASD.

## Author contributions

PS: Conceptualization, Funding acquisition, Writing – original draft, Writing – review & editing. AP: Writing – original draft, Writing – review & editing. JL: Conceptualization, Funding acquisition, Writing – original draft, Writing – review & editing. AA: Writing – original draft, Writing – review & editing, Conceptualization.

## References

[ref1] Adiletta A. Pedrana S. Rosa-Salva O. Sgadò P. (2021). Spontaneous visual preference for face-like stimuli is impaired in newly-hatched domestic chicks exposed to Valproic acid during embryogenesis. Front. Behav. Neurosci. 15:733140. doi: 10.3389/fnbeh.2021.733140, PMID: 34858146 PMC8632556

[ref2] Adiletta A. Pross A. Taricco N. Sgadò P. (2022). Embryonic valproate exposure alters mesencephalic dopaminergic neurons distribution and septal dopaminergic gene expression in domestic chicks. Front. Integr. Neurosci. 16:804881. doi: 10.3389/fnint.2022.804881, PMID: 35369647 PMC8966611

[ref3] Almasi R. C. Behrmann M. (2021). Subcortical regions of the human visual system do not process faces holistically. Brain Cogn. 151:105726. doi: 10.1016/j.bandc.2021.105726, PMID: 33933856 PMC8194320

[ref4] Avarguès-Weber A. d’Amaro D. Metzler M. Finke V. Baracchi D. Dyer A. G. (2018). Does holistic processing require a large brain? Insights from honeybees and wasps in fine visual recognition tasks. Front. Psychol. 9:1313. doi: 10.3389/fpsyg.2018.01313, PMID: 30108535 PMC6079261

[ref5] Barber A. L. A. Randi D. Müller C. A. Huber L. (2016). The processing of human emotional faces by pet and lab dogs: evidence for lateralization and experience effects. PLoS One 11:e0152393. doi: 10.1371/journal.pone.0152393, PMID: 27074009 PMC4830442

[ref6] Bathelt J. Koolschijn P. C. M. Geurts H. M. (2022). Atypically slow processing of faces and non-faces in older autistic adults. Autism 26, 1737–1751. doi: 10.1177/13623613211065297, PMID: 34961340 PMC9483195

[ref7] Bauman M. D. Schumann C. M. (2018). Advances in nonhuman primate models of autism: integrating neuroscience and behavior. Exp. Neurol. 299, 252–265. doi: 10.1016/j.expneurol.2017.07.021, PMID: 28774750 PMC5810939

[ref8] Bi T. Fang F. (2017). Impaired face perception in individuals with autism Spectrum disorder: insights on diagnosis and treatment. Neurosci. Bull. 33, 757–759. doi: 10.1007/s12264-017-0187-1, PMID: 29119345 PMC5725389

[ref9] Bortolon C. Capdevielle D. Raffard S. (2015). Face recognition in schizophrenia disorder: a comprehensive review of behavioral, neuroimaging and neurophysiological studies. Neurosci. Biobehav. Rev. 53, 79–107. doi: 10.1016/j.neubiorev.2015.03.006, PMID: 25800172

[ref10] Bradshaw J. Klin A. Evans L. Klaiman C. Saulnier C. McCracken C. (2020). Development of attention from birth to 5 months in infants at risk for autism spectrum disorder. Dev. Psychopathol. 32, 491–501. doi: 10.1017/s0954579419000233, PMID: 31012398 PMC6812597

[ref11] Brecht K. F. Wagener L. Ostojić L. Clayton N. S. Nieder A. (2017). Comparing the face inversion effect in crows and humans. J. Comp. Physiol. A. 203, 1017–1027. doi: 10.1007/s00359-017-1211-7, PMID: 28905251 PMC5696503

[ref12] Buiatti M. Giorgio E. D. Piazza M. Polloni C. Menna G. Taddei F.. (2019). Cortical route for facelike pattern processing in human newborns. Proc. Natl. Acad. Sci. 116, 4625–4630. doi: 10.1073/pnas.1812419116, PMID: 30755519 PMC6410830

[ref13] Calder A. J. Young A. W. (2005). Understanding the recognition of facial identity and facial expression. Nat. Rev. Neurosci. 6, 641–651. doi: 10.1038/nrn1724 16062171

[ref14] Campatelli G. Federico R. R. Apicella F. Sicca F. Muratori F. (2013). Face processing in children with ASD: literature review. Res. Autism Spectr. Disord. 7, 444–454. doi: 10.1016/j.rasd.2012.10.003

[ref15] Carver L. J. Dawson G. (2002). Development and neural bases of face recognition in autism. Mol. Psychiatry 7, S18–S20. doi: 10.1038/sj.mp.4001168, PMID: 12142937

[ref16] Chan A. Trefler A. Babajani-Feremi A. (2016). Verifying face selectivity in the human prefrontal cortex: data from \textbackslashtextasciitilde500 participants. J. Vis. 16:718. doi: 10.1167/16.12.718

[ref17] Chen Y. Norton D. Ongur D. Heckers S. (2008). Inefficient face detection in schizophrenia. Schizophr. Bull. 34, 367–374. doi: 10.1093/schbul/sbm071, PMID: 17631619 PMC2632392

[ref18] Chita-Tegmark M. (2016). Social attention in ASD: a review and meta-analysis of eye-tracking studies. Res. Dev. Disabil. 48, 79–93. doi: 10.1016/j.ridd.2015.10.011, PMID: 26547134

[ref19] Christensen J. Grønborg T. Sørensen M. Schendel D. Parner E. Pedersen L.. (2013). Prenatal valproate exposure and risk of autism spectrum disorders and childhood autism. JAMA 309, 1696–1703. doi: 10.1001/jama.2013.2270, PMID: 23613074 PMC4511955

[ref20] Clark W. Chilcott M. Azizi A. Pusch R. Perry K. Colombo M. (2022). Neurons in the pigeon visual network discriminate between faces, scrambled faces, and sine grating images. Sci. Rep. 12:589. doi: 10.1038/s41598-021-04559-z, PMID: 35022466 PMC8755821

[ref21] Collin L. Bindra J. Raju M. Gillberg C. Minnis H. (2013). Facial emotion recognition in child psychiatry: a systematic review. Res. Dev. Disabil. 34, 1505–1520. doi: 10.1016/j.ridd.2013.01.008, PMID: 23475001

[ref22] Csillag A. Ádám Á. Zachar G. (2022). Avian models for brain mechanisms underlying altered social behavior in autism. Front. Physiol. 13:1032046. doi: 10.3389/fphys.2022.1032046, PMID: 36388132 PMC9650632

[ref23] Cuaya L. V. Hernández-Pérez R. Concha L. (2016). Our faces in the Dog’s brain: functional imaging reveals temporal cortex activation during perception of human faces. PLoS One 11:e0149431. doi: 10.1371/journal.pone.0149431, PMID: 26934715 PMC4774982

[ref24] Dalrymple K. A. Khan A. F. Duchaine B. Elison J. T. (2021). Visual input to the left versus right eye yields differences in face preferences in 3-month-old infants. Dev. Sci. 24:e13029. doi: 10.1111/desc.13029, PMID: 32772413 PMC7870722

[ref25] Dawson G. Webb S. J. McPartland J. (2005). Understanding the nature of face processing impairment in autism: insights from behavioral and electrophysiological studies. Dev. Neuropsychol. 27, 403–424. doi: 10.1207/s15326942dn2703_6, PMID: 15843104

[ref26] Di Giorgio E. Frasnelli E. Salva O. R. Luisa S. M. Puopolo M. Tosoni D.. (2016). Difference in visual social predispositions between newborns at low-and high-risk for autism. Sci. Rep. 6:26395. doi: 10.1038/srep26395, PMID: 27198160 PMC4873740

[ref27] Di Giorgio E. Loveland J. L. Mayer U. Rosa-Salva O. Versace E. Vallortigara G. (2017). Filial responses as predisposed and learned preferences: early attachment in chicks and babies. Behav. Brain Res. 325, 90–104. doi: 10.1016/j.bbr.2016.09.018 27616345

[ref28] Di Giorgio E. Rosa-Salva O. Frasnelli E. Calcagnì A. Lunghi M. Scattoni M. L.. (2021). Abnormal visual attention to simple social stimuli in 4-month-old infants at high risk for autism. Sci. Rep. 11:15785. doi: 10.1038/s41598-021-95418-4, PMID: 34349200 PMC8338945

[ref29] Djurdjevic V. Ansuini A. Bertolini D. Macke J. Zoccolan D. (2018). Accuracy of rats in discriminating visual objects is explained by the complexity of their perceptual strategy. Curr. Biol. 28, 1005–1015.e5. doi: 10.1016/j.cub.2018.02.037 29551414 PMC5887110

[ref30] Duchaine B. Yovel G. (2015). A revised neural framework for face processing. Ann. Rev. Vision Sci. 1, 393–416. doi: 10.1146/annurev-vision-082114-035518, PMID: 28532371

[ref31] Farroni T. Johnson M. H. Menon E. Zulian L. Faraguna D. Csibra G. (2005). Newborns’ preference for face-relevant stimuli: effects of contrast polarity. Proc. Natl. Acad. Sci. 102, 17245–17250. doi: 10.1073/pnas.0502205102, PMID: 16284255 PMC1287965

[ref32] Fujita K. (1987). Species recognition by five macaque monkeys. Primates 28, 353–366. doi: 10.1007/bf02381018

[ref33] Fujita K. (1993). Development of visual preference for closely related species by infant and juvenile macaques with restricted social experience. Primates 34, 141–150. doi: 10.1007/bf02381385

[ref34] Gandhi S. Piacentino M. Vieceli F. Bronner M. (2017). Optimization of CRISPR/Cas9 genome editing for loss-of-function in the early chick embryo. Dev. Biol. 432, 86–97. doi: 10.1016/j.ydbio.2017.08.036 29150011 PMC5728388

[ref35] Gauthier I. Tarr M. J. Moylan J. Skudlarski P. Gore J. C. Anderson A. W. (2000). The fusiform face area is part of a network that processes faces at the individual level. J. Cogn. Neurosci. 12, 495–504. doi: 10.1162/089892900562165 10931774

[ref36] Goren C. Sarty M. Wu P. (1975). Visual following and pattern discrimination of face-like stimuli by newborn infants. Pediatrics 56, 544–549. doi: 10.1542/peds.56.4.544 1165958

[ref37] Guerra M. Medici V. Weatheritt R. Corvino V. Palacios D. Geloso M. C.. (2023). Fetal exposure to valproic acid dysregulates the expression of autism-linked genes in the developing cerebellum. Transl. Psychiatry 13:114. doi: 10.1038/s41398-023-02391-9, PMID: 37019889 PMC10076313

[ref38] Haxby J. V. Hoffman E. A. Gobbini M. I. Haxby J. V. Hoffman E. A. Gobbini M. I. (2000). The distributed human neural system for face perception. Trends Cogn. Sci. 4, 223–233. doi: 10.1016/s1364-6613(00)01482-0 10827445

[ref39] Heekeren H. R. Marrett S. Bandettini P. A. Ungerleider L. G. (2004). A general mechanism for perceptual decision-making in the human brain. Nature 431, 859–862. doi: 10.1038/nature02966 15483614

[ref40] Hoffman E. A. Haxby J. V. (2000). Distinct representations of eye gaze and identity in the distributed human neural system for face perception. Nat. Neurosci. 3, 80–84. doi: 10.1038/71152, PMID: 10607399

[ref41] Huang Y. Vangel M. Chen H. Eshel M. Cheng M. Lu T.. (2022). The impaired subcortical pathway from superior colliculus to the amygdala in boys with autism Spectrum disorder. Front. Integr. Neurosci. 16:666439. doi: 10.3389/fnint.2022.666439, PMID: 35784498 PMC9247550

[ref42] Hung C.-C. Yen C. C. Ciuchta J. L. Papoti D. Bock N. A. Leopold D. A.. (2015). Functional mapping of face-selective regions in the extrastriate visual cortex of the marmoset. J. Neurosci. 35, 1160–1172. doi: 10.1523/jneurosci.2659-14.2015, PMID: 25609630 PMC4300322

[ref43] Johnson M. H. (2005). Subcortical face processing. Nat. Rev. Neurosci. 6, 766–774. doi: 10.1038/nrn1766 16276354

[ref44] Johnson M. H. (2014). Autism: demise of the innate social orienting hypothesis. Curr. Biol. 24, R30–R31. doi: 10.1016/j.cub.2013.11.021 24405675

[ref45] Johnson M. Dziurawiec S. Ellis H. Morton J. (1991). Newborns’ preferential tracking of face-like stimuli and its subsequent decline. Cognition 40, 1–19. doi: 10.1016/0010-0277(91)90045-6 1786670

[ref46] Johnson M. H. Senju A. Tomalski P. (2015). The two-process theory of face processing: modifications based on two decades of data from infants and adults. Neurosci. Biobehav. Rev. 50, 169–179. doi: 10.1016/j.neubiorev.2014.10.009, PMID: 25454353

[ref47] Kanwisher N. McDermott J. Chun M. M. (1997). The fusiform face area: a module in human extrastriate cortex specialized for face perception. J. Neurosci. Off. J. Soc. Neurosci. 17, 4302–4311. doi: 10.1523/JNEUROSCI.17-11-04302.1997, PMID: 9151747 PMC6573547

[ref48] Karplus I. Algom D. (1981). Visual cues for predator face recognition by reef fishes. Z. Tierpsychol. 55, 343–364. doi: 10.1111/j.1439-0310.1981.tb01277.x

[ref49] Katsnelson A. (2018). Modeling autism. Lab Anim. 47, 41–44. doi: 10.1038/laban.1394 29384512

[ref50] Kleiner K. A. (1987). Amplitude and phase spectra as indices of infants’ pattern preferences. Infant Behav. Dev. 10, 49–59. doi: 10.1016/0163-6383(87)90006-3

[ref51] Kleinhans N. M. Richards T. Johnson L. C. Weaver K. E. Greenson J. Dawson G.. (2011). fMRI evidence of neural abnormalities in the subcortical face processing system in ASD. NeuroImage 54, 697–704. doi: 10.1016/j.neuroimage.2010.07.037, PMID: 20656041 PMC3426450

[ref52] Kliemann D. Dziobek I. Hatri A. Baudewig J. Heekeren H. R. (2012). The role of the amygdala in atypical gaze on emotional faces in autism Spectrum disorders. J. Neurosci. 32, 9469–9476. doi: 10.1523/jneurosci.5294-11.2012, PMID: 22787032 PMC6622276

[ref53] Klin A. Jones W. Schultz R. Volkmar F. Cohen D. (2002). Visual fixation patterns during viewing of naturalistic social situations as predictors of social competence in individuals with autism. Arch. Gen. Psychiatry 59, 809–816. doi: 10.1001/archpsyc.59.9.809, PMID: 12215080

[ref54] Kobylkov D. Rosa-Salva O. Zanon M. Vallortigara G. (2024). Innate face detectors in the nidopallium of young domestic chicks. bioRxiv:2024.02.15.580445. doi: 10.1101/2024.02.15.580445

[ref55] Kobylkov D. Vallortigara G. (2024). Face detection mechanisms: Nature vs. nurture. Front. Neurosci. 18:1404174. doi: 10.3389/fnins.2024.1404174, PMID: 38812973 PMC11133589

[ref56] Krueger B. Dorsey S. Mocci E. Lane M. (2024). Rapid effects of valproic acid on the fetal brain transcriptome: implications for brain development and autism. Research Square. [Preprint]. doi: 10.21203/rs.3.rs-3684653/v1 PMC11618798 39632793

[ref57] Lamanna J. Meldolesi J. (2024). Autism Spectrum disorder: brain areas involved, neurobiological mechanisms, diagnoses and therapies. Int. J. Mol. Sci. 25:2423. doi: 10.3390/ijms25042423 38397100 PMC10889781

[ref58] Le Q. V. Le Q. V. Nishimaru H. Matsumoto J. Takamura Y. Hori E.. (2020). A prototypical template for rapid face detection is embedded in the monkey superior colliculus. Front. Syst. Neurosci. 14:5. doi: 10.3389/fnsys.2020.00005, PMID: 32158382 PMC7025518

[ref59] Liu J. Harris A. Kanwisher N. (2010). Perception of face parts and face configurations: an fMRI study. J. Cogn. Neurosci. 22, 203–211. doi: 10.1162/jocn.2009.21203, PMID: 19302006 PMC2888696

[ref60] Lorenzi E. Pross A. Rosa-Salva O. Versace E. Sgadò P. Vallortigara G. (2019). Embryonic exposure to Valproic acid affects social predispositions for dynamic cues of animate motion in newly-hatched chicks. Front. Physiol. 10:501. doi: 10.3389/fphys.2019.00501, PMID: 31114510 PMC6503819

[ref61] Loth E. Garrido L. Ahmad J. Watson E. Duff A. Duchaine B. (2018). Facial expression recognition as a candidate marker for autism spectrum disorder: how frequent and severe are deficits? Mol. Autism 9:7. doi: 10.1186/s13229-018-0187-7 PMC5791186 29423133

[ref62] Lozier L. M. Vanmeter J. W. Marsh A. A. (2014). Impairments in facial affect recognition associated with autism spectrum disorders: a meta-analysis. Dev. Psychopathol. 26, 933–945. doi: 10.1017/s0954579414000479 24915526

[ref63] Matsushima T. Izumi T. Vallortigara G. (2024). The domestic chick as an animal model of autism spectrum disorder: building adaptive social perceptions through prenatally formed predispositions. Front. Neurosci. 18:1279947. doi: 10.3389/fnins.2024.1279947, PMID: 38356650 PMC10864568

[ref64] McCarthy G. Puce A. Gore J. C. Allison T. (1997). Face-specific processing in the human fusiform gyrus. J. Cogn. Neurosci. 9, 605–610. doi: 10.1162/jocn.1997.9.5.605 23965119

[ref65] Mende-Siedlecki P. Verosky S. C. Turk-Browne N. B. Todorov A. (2013). Robust selectivity for faces in the human amygdala in the absence of expressions. J. Cogn. Neurosci. 25, 2086–2106. doi: 10.1162/jocn_a_00469, PMID: 23984945 PMC4029947

[ref66] Meng Q. Zhang W. Wang X. Jiao C. Xu S. Liu C.. (2022). Human forebrain organoids reveal connections between valproic acid exposure and autism risk. Transl. Psychiatry 12:130. doi: 10.1038/s41398-022-01898-x, PMID: 35351869 PMC8964691

[ref67] Minio-Paluello I. Porciello G. Pascual-Leone A. Baron-Cohen S. (2020). Face individual identity recognition: a potential endophenotype in autism. Mol. Autism. 11:81. doi: 10.1186/s13229-020-00371-0, PMID: 33081830 PMC7576748

[ref68] Morton J. Johnson M. H. (1991). CONSPEC and CONLERN: a two-process theory of infant face recognition. Psychol. Rev. 98, 164–181. doi: 10.1037/0033-295x.98.2.164, PMID: 2047512

[ref69] Nakagami A. Yasue M. Nakagaki K. Nakamura M. Kawai N. Ichinohe N. (2022). Reduced childhood social attention in autism model marmosets predicts impaired social skills and inflexible behavior in adulthood. Front. Psych. 13:885433. doi: 10.3389/fpsyt.2022.885433, PMID: 35958665 PMC9357878

[ref70] Nickl-Jockschat T. Rottschy C. Thommes J. Schneider F. Laird A. R. Fox P. T.. (2015). Neural networks related to dysfunctional face processing in autism spectrum disorder. Brain Struct. Funct. 220, 2355–2371. doi: 10.1007/s00429-014-0791-z, PMID: 24869925 PMC4782795

[ref71] Nunes A. R. Carreira L. Anbalagan S. Blechman J. Levkowitz G. Oliveira R. F. (2020). Perceptual mechanisms of social affiliation in zebrafish. Sci. Rep. 10:3642. doi: 10.1038/s41598-020-60154-8 32107434 PMC7046791

[ref72] Parr L. A. Hecht E. Barks S. K. Preuss T. M. Votaw J. R. (2009). Face processing in the chimpanzee brain. Curr. Biol. 19, 50–53. doi: 10.1016/j.cub.2008.11.048 19097899 PMC2651677

[ref73] Pascalis O. Damon F. Guo K. Méary D. (2021). It takes one to know one: do human and nonhuman Primates share similar face processing? Singapore: Springer Singapore.

[ref74] Pascalis O. Kelly D. J. (2009). The origins of face processing in humans: phylogeny and ontogeny. Perspect. Psychol. Sci. 4, 200–209. doi: 10.1111/j.1745-6924.2009.01119.x, PMID: 26158945

[ref75] Pavlova M. A. Guerreschi M. Tagliavento L. Gitti F. Sokolov A. N. Fallgatter A. J.. (2017). Social cognition in autism: face tuning. Sci. Rep. 7:2734. doi: 10.1038/s41598-017-02790-1, PMID: 28578379 PMC5457440

[ref76] Pelphrey K. A. Sasson N. J. Reznick J. S. Paul G. Goldman B. D. Piven J. (2002). Visual scanning of faces in autism. J. Autism Dev. Disord. 32, 249–261. doi: 10.1023/a:1016374617369 12199131

[ref77] Pessoa L. Adolphs R. (2010). Emotion processing and the amygdala: from a “low road” to “many roads” of evaluating biological significance. Nat. Rev. Neurosci. 11, 773–782. doi: 10.1038/nrn2920, PMID: 20959860 PMC3025529

[ref78] Philip R. C. M. Dauvermann M. R. Whalley H. C. Baynham K. Lawrie S. M. Stanfield A. C. (2012). A systematic review and meta-analysis of the fMRI investigation of autism spectrum disorders. Neurosci. Biobehav. Rev. 36, 901–942. doi: 10.1016/j.neubiorev.2011.10.008 22101112

[ref79] Pitcher D. Walsh V. Yovel G. Duchaine B. (2007). TMS evidence for the involvement of the right occipital face area in early face processing. Curr. Biol. 17, 1568–1573. doi: 10.1016/j.cub.2007.07.063, PMID: 17764942

[ref80] Proops L. McComb K. (2012). Cross-modal individual recognition in domestic horses (*Equus caballus*) extends to familiar humans. Proc. R. Soc. B Biol. Sci. 279, 3131–3138. doi: 10.1098/rspb.2012.0626, PMID: 22593108 PMC3385734

[ref81] Reid V. Dunn K. Young R. Amu J. Donovan T. Reissland N. (2017). The human fetus preferentially engages with face-like visual stimuli. Curr. Biol. 27, 1825–1828.e3. doi: 10.1016/j.cub.2017.05.044 28602654

[ref82] Reisinger D. L. Shaffer R. C. Horn P. S. Hong M. P. Pedapati E. V. Dominick K. C.. (2020). Atypical social attention and emotional face processing in autism Spectrum disorder: insights from face scanning and Pupillometry. Front. Integr. Neurosci. 13:76. doi: 10.3389/fnint.2019.00076, PMID: 32116580 PMC7026501

[ref83] Renzi C. Schiavi S. Carbon C.-C. Vecchi T. Silvanto J. Cattaneo Z. (2013). Processing of featural and configural aspects of faces is lateralized in dorsolateral prefrontal cortex: a TMS study. NeuroImage 74, 45–51. doi: 10.1016/j.neuroimage.2013.02.015, PMID: 23435211

[ref84] Riby D. M. Hancock P. J. B. (2009). Do faces capture the attention of individuals with Williams syndrome or autism? Evidence from tracking eye movements. J. Autism Dev. Disord. 39, 421–431. doi: 10.1007/s10803-008-0641-z 18787936

[ref85] Rieblinger B. Sid H. Duda D. Bozoglu T. Klinger R. Schlickenrieder A.. (2021). Cas9-expressing chickens and pigs as resources for genome editing in livestock. Proc. Natl. Acad. Sci. U. S. A. 118:e2022562118. doi: 10.1073/pnas.2022562118, PMID: 33658378 PMC7958376

[ref86] Romagnano V. Sokolov A. N. Steinwand P. Fallgatter A. J. Pavlova M. A. (2022). Face pareidolia in male schizophrenia. Schizophrenia 8:112. doi: 10.1038/s41537-022-00315-y, PMID: 36517504 PMC9751144

[ref87] Rosa Salva O. Mayer U. Vallortigara G. (2015). Roots of a social brain: developmental models of emerging animacy-detection mechanisms. Neurosci. Biobehav. Rev. 50, 150–168. doi: 10.1016/j.neubiorev.2014.12.015, PMID: 25544151

[ref88] Rosa Salva O. Regolin L. Vallortigara G. (2012). Inversion of contrast polarity abolishes spontaneous preferences for face-like stimuli in newborn chicks. Behav. Brain Res. 228, 133–143. doi: 10.1016/j.bbr.2011.11.025, PMID: 22155610

[ref89] Rosa-Salva O. Regolin L. Vallortigara G. (2010). Faces are special for newly hatched chicks: evidence for inborn domain-specific mechanisms underlying spontaneous preferences for face-like stimuli. Dev. Sci. 13, 565–577. doi: 10.1111/j.1467-7687.2009.00914.x, PMID: 20590721

[ref90] Rossion B. Taubert J. (2019). What can we learn about human individual face recognition from experimental studies in monkeys? Vis. Res. 157, 142–158. doi: 10.1016/j.visres.2018.03.012 31230664

[ref91] Russell M. T. Hajdúk M. Springfield C. R. Klein H. S. Bass E. L. Mittal V. A.. (2024). Identity recognition from faces and bodies in schizophrenia spectrum disorders. Schizophrenia Res. Cognit. 36:100307. doi: 10.1016/j.scog.2024.100307, PMID: 38486791 PMC10937230

[ref92] Rutishauser U. Tudusciuc O. Wang S. Mamelak A. N. Ross I. B. Adolphs R. (2013). Single-neuron correlates of atypical face processing in autism. Neuron 80, 887–899. doi: 10.1016/j.neuron.2013.08.029, PMID: 24267649 PMC3934412

[ref93] Salter D. W. Smith E. J. Hughes S. H. Wright S. E. Crittenden L. B. (1987). Transgenic chickens: insertion of retroviral genes into the chicken germ line. Virology 157, 236–240. doi: 10.1016/0042-6822(87)90334-5 3029962

[ref94] Schaeffer D. J. Selvanayagam J. Johnston K. D. Menon R. S. Freiwald W. A. Everling S. (2020). Face selective patches in marmoset frontal cortex. Nat. Commun. 11:4856. doi: 10.1038/s41467-020-18692-2, PMID: 32978385 PMC7519082

[ref95] Schnell A. Van den Bergh G. Vermaercke B. Gijbels K. Bossens C. de Beeck H. (2019). Face categorization and behavioral templates in rats. J. Vis. 19:9. doi: 10.1167/19.14.9, PMID: 31826254

[ref96] Schultz R. T. Gauthier I. Klin A. Fulbright R. K. Anderson A. W. Volkmar F.. (2000). Abnormal ventral temporal cortical activity during face discrimination among individuals with autism and Asperger syndrome. Arch. Gen. Psychiatry 57, 331–340. doi: 10.1001/archpsyc.57.4.331 10768694

[ref97] Schultz R. T. Grelotti D. J. Klin A. Kleinman J. der Gaag C. V. Marois R.. (2003). The role of the fusiform face area in social cognition: implications for the pathobiology of autism. Philosophical transactions of the Royal Society of London. Series B Biol. Sci. 358, 415–427. doi: 10.1098/rstb.2002.1208, PMID: 12639338 PMC1693125

[ref98] Schultz R. T. Klin A. (2002). Genetics of childhood disorders: XLIII. Autism, part 2: neural foundations. J. Am. Acad. Child Adolesc. Psychiatry 41, 1259–1262. doi: 10.1097/00004583-200210000-00018, PMID: 12364850

[ref99] Sgadò P. Rosa-Salva O. Versace E. Vallortigara G. (2018). Embryonic exposure to Valproic acid impairs social predispositions of newly-hatched chicks. Sci. Rep. 8:5919. doi: 10.1038/s41598-018-24202-8, PMID: 29650996 PMC5897402

[ref100] Shephard E. Milosavljevic B. Mason L. Elsabbagh M. Tye C. Gliga T.. (2020). Neural and behavioural indices of face processing in siblings of children with autism spectrum disorder (ASD): a longitudinal study from infancy to mid-childhood. Cortex 127, 162–179. doi: 10.1016/j.cortex.2020.02.008, PMID: 32200288 PMC7254063

[ref101] Sid H. Schusser B. (2018). Applications of gene editing in chickens: a new era is on the horizon. Front. Genet. 9:456. doi: 10.3389/fgene.2018.00456, PMID: 30356667 PMC6189320

[ref102] Simion F. Di Giorgio E. (2015). Face perception and processing in early infancy: inborn predispositions and developmental changes. Front. Psychol. 6:969. doi: 10.3389/fpsyg.2015.00969 26217285 PMC4496551

[ref103] Simpson E. A. Jakobsen K. V. Damon F. Suomi S. J. Ferrari P. F. Paukner A. (2017). Face detection and the development of own-species Bias in infant macaques. Child Dev. 88, 103–113. doi: 10.1111/cdev.12565, PMID: 27223687 PMC5123966

[ref104] Sugita Y. (2008). Face perception in monkeys reared with no exposure to faces. Proc. Natl. Acad. Sci. USA 105, 394–398. doi: 10.1073/pnas.0706079105, PMID: 18172214 PMC2224224

[ref105] Sugita Y. (2009). Innate face processing. Curr. Opin. Neurobiol. 19, 39–44. doi: 10.1016/j.conb.2009.03.001 19339171

[ref106] Suwandschieff E. Mundry R. Kull K. Kreuzer L. Schwing R. (2023). ‘Do I know you?’ Categorizing individuals on the basis of familiarity in kea (*Nestor notabilis*). R. Soc. Open Sci. 10:230228. doi: 10.1098/rsos.230228, PMID: 37351495 PMC10282571

[ref107] Thompson P. (1980). Margaret Thatcher: a new illusion. Perception 9, 483–484. doi: 10.1068/p090483 6999452

[ref108] Tibbetts E. A. Pardo-Sanchez J. Ramirez-Matias J. Avarguès-Weber A. (2021). Individual recognition is associated with holistic face processing in Polistes paper wasps in a species-specific way. Proc. R. Soc. B 288:20203010. doi: 10.1098/rspb.2020.3010, PMID: 33468004 PMC7893282

[ref109] Tong F. Nakayama K. Moscovitch M. Weinrib O. Kanwisher N. (2000). Response properties of the human fusiform face area. Cogn. Neuropsychol. 17, 257–280. doi: 10.1080/026432900380607 20945183

[ref110] Towler A. Kemp R. I. Bruce V. Burton A. M. Dunn J. D. White D. (2019). Are face recognition abilities in humans and sheep really ‘comparable’? R. Soc. Open Sci. 6:180772. doi: 10.1098/rsos.180772, PMID: 30800343 PMC6366218

[ref111] Tsao D. Y. Livingstone M. S. (2008). Mechanisms of face perception. Annu. Rev. Neurosci. 31, 411–437. doi: 10.1146/annurev.neuro.30.051606.094238, PMID: 18558862 PMC2629401

[ref112] Tsao D. Y. Moeller S. Freiwald W. A. (2008). Comparing face patch systems in macaques and humans. Proc. Natl. Acad. Sci. 105, 19514–19519. doi: 10.1073/pnas.0809662105, PMID: 19033466 PMC2614792

[ref113] Turati C. Simion F. Milani I. Umiltà C. (2002). Newborns’ preference for faces: what is crucial? Dev. Psychol. 38, 875–882. doi: 10.1037/0012-1649.38.6.875 12428700

[ref114] Tye C. Bussu G. Gliga T. Elsabbagh M. Pasco G. Johnsen K.. (2022). Understanding the nature of face processing in early autism: a prospective study. J. Psychopathol. Clin. Sci. 131, 542–555. doi: 10.1037/abn0000648, PMID: 35901386 PMC9330670

[ref115] Uljarevic M. Hamilton A. (2013). Recognition of emotions in autism: a formal Meta-analysis. J. Autism Dev. Disord. 43, 1517–1526. doi: 10.1007/s10803-012-1695-5 23114566

[ref116] Valenza E. Simion F. Cassia V. M. Umiltà C. (1996). Face preference at birth. J. Exp. Psychol. Hum. Percept. Perform. 22, 892–903. doi: 10.1037/0096-1523.22.4.892 8756957

[ref117] Vallortigara G. Regolin L. Marconato F. (2005). Visually inexperienced chicks exhibit spontaneous preference for biological motion patterns. PLoS Biol. 3:e208. doi: 10.1371/journal.pbio.0030208, PMID: 15934787 PMC1150290

[ref118] Versace E. Damini S. Stancher G. (2020). Early preference for face-like stimuli in solitary species as revealed by tortoise hatchlings. Proc. Natl. Acad. Sci. U. S. A. 117, 24047–24049. doi: 10.1073/pnas.2011453117, PMID: 32929003 PMC7533828

[ref119] Wagner J. B. Luyster R. J. Yim J. Y. Tager-Flusberg H. Nelson C. A. (2013). The role of early visual attention in social development. Int. J. Behav. Dev. 37, 118–124. doi: 10.1177/0165025412468064, PMID: 26478642 PMC4606456

[ref120] Wang Y. Cao R. Chakravarthula P. N. Yu H. Wang S. (2024). Atypical neural encoding of faces in individuals with autism spectrum disorder. Cereb. Cortex 34, 172–186. doi: 10.1093/cercor/bhae060, PMID: 38696606 PMC11065108

[ref121] Wang S. Li X. (2023). A revisit of the amygdala theory of autism: twenty years after. Neuropsychologia 183:108519. doi: 10.1016/j.neuropsychologia.2023.108519, PMID: 36803966 PMC10824605

[ref122] Wang M.-Y. Takeuchi H. (2017). Individual recognition and the ‘face inversion effect’ in medaka fish (*Oryzias latipes*). eLife 6:e24728. doi: 10.7554/elife.24728, PMID: 28693720 PMC5505697

[ref123] Watanabe S. Masuda S. Shinozuka K. Borlongan C. (2022). Preference and discrimination of facial expressions of humans, rats, and mice by C57 mice. Anim. Cogn. 25, 297–306. doi: 10.1007/s10071-021-01551-y, PMID: 34417921

[ref124] Watson K. K. Platt M. L. (2012). Of mice and monkeys: using non-human primate models to bridge mouse-and human-based investigations of autism spectrum disorders. J. Neurodev. Disord. 4:21. doi: 10.1186/1866-1955-4-21 22958282 PMC3445833

[ref125] Webb S. Neuhaus E. Faja S. (2017). Face perception and learning in autism spectrum disorders. Q. J. Exp. Psychol. 70, 970–986. doi: 10.1080/17470218.2016.1151059, PMID: 26886246 PMC5026554

[ref126] Weigelt S. Koldewyn K. Kanwisher N. (2012). Face identity recognition in autism spectrum disorders: a review of behavioral studies. Neurosci. Biobehav. Rev. 36, 1060–1084. doi: 10.1016/j.neubiorev.2011.12.008 22212588

[ref127] Yovel G. Kanwisher N. (2005). The neural basis of the behavioral face-inversion effect. Curr. Biol. 15, 2256–2262. doi: 10.1016/j.cub.2005.10.072, PMID: 16360687

[ref128] Zangenehpour S. Chaudhuri A. (2005). Patchy organization and asymmetric distribution of the neural correlates of face processing in monkey Inferotemporal cortex. Curr. Biol. 15, 993–1005. doi: 10.1016/j.cub.2005.04.031 15936269

[ref129] Zanon M. Lemaire B. S. Papeo L. Vallortigara G. (2024). Innate sensitivity to face-to-face biological motion. iScience 27:108793. doi: 10.1016/j.isci.2024.108793 38299110 PMC10828802

[ref130] Zarate-Lopez D. Torres-Chávez A. L. Gálvez-Contreras A. Y. Gonzalez-Perez O. (2024). Three decades of valproate: a current model for studying autism Spectrum disorder. Curr. Neuropharmacol. 22, 260–289. doi: 10.2174/1570159x22666231003121513, PMID: 37873949 PMC10788883

[ref131] Zhao H. Jiang Y. Zhang Y. Q. (2018). Modeling autism in non-human primates: opportunities and challenges. Autism Res. 11, 686–694. doi: 10.1002/aur.1945, PMID: 29573234 PMC6188783

[ref132] Zhao H. Wang Q. Yan T. Zhang Y. Xu H. Yu H.. (2019). Maternal valproic acid exposure leads to neurogenesis defects and autism-like behaviors in non-human primates. Transl. Psychiatry 9:267. doi: 10.1038/s41398-019-0608-1, PMID: 31636273 PMC6803711

[ref133] Zhou Y. Sharma J. Ke Q. Landman R. Yuan J. Chen H.. (2019). Atypical behaviour and connectivity in SHANK3-mutant macaques. Nature 570, 326–331. doi: 10.1038/s41586-019-1278-0, PMID: 31189958

